# The Transient Receptor Potential Vanilloid 2 Cation Channel Is Abundant in Macrophages Accumulating at the Peri-Infarct Zone and May Enhance Their Migration Capacity towards Injured Cardiomyocytes following Myocardial Infarction

**DOI:** 10.1371/journal.pone.0105055

**Published:** 2014-08-19

**Authors:** Michal Entin-Meer, Ran Levy, Pavel Goryainov, Natalie Landa, Iris Barshack, Camila Avivi, Jonathan Semo, Gad Keren

**Affiliations:** 1 Laboratory of Cardiovascular Research, Department of Cardiology, Tel-Aviv Sourasky Medical Center, Tel-Aviv, Israel; 2 Sackler School of Medicine, Tel-Aviv University, Tel Aviv, Israel; 3 Neufeld Cardiac Research Institute, Sheba Medical Center, Ramat Gan, Israel; 4 Department of Pathology, Sheba Medical Center, Ramat Gan, Israel; University of Central Florida, United States of America

## Abstract

**Purpose:**

A novel family of transient receptor potential (TRP) channels, that may hold a role in calcium homeostasis, has recently been described. By employing a GeneChip array analysis we have demonstrated a clear and specific upregulation of the TRP vanilloid 2 (TRPV2) mRNA in the left ventricles (LV) 3–5 days post-acute myocardial infarction (MI) compared to sham-operated controls, both in rats and in mice. We sought to characterize the cardiac cellular subpopulations in which TRPV2 is overexpressed upon acute MI.

**Methods:**

Lewis rats underwent an acute MI by ligation of the left anterior descending artery or chest opening only (sham). The animals were terminated at various time points and an immunohistochemical (IHC) and immunofloerescent (IFC) staining of the LV sections as well as a flow cytometry analysis of LV-derived cells were carried out, using anti-TRPV2 and anti-monocyte/macrophage antibodies. Rat alveolar macrophage cells, NR8383, transiently transfected with TRPV2 siRNA were allowed to migrate towards hypoxic conditioned media of the rat cardiac myoblast line H9C2 using a trans-well migration assay. The macrophage cells migrating to the bottom side of the inserts were counted.

**Results:**

The IHC and IFC staining as well as the flow cytometry data demonstrated a substantial expression of TRPV2 in infiltrating macrophages in the peri-infarct region 3–5 days post-acute MI. The in vitro migration assay data demonstrated that following inhibition of the TRPV2 channel, the number of migrating macrophages towards conditioned medium of hypoxic cardiomyocytes was significantly reduced.

**Conclusions:**

TRPV2 is highly expressed on the peri-infarct infiltrating macrophages and may play an important role in post-MI phagocytosis. Better characterization of this channel may pave the way for identifying a new target for modulating the dramatic post-MI immune reactions.

## Introduction

Myocardial infarction and subsequent development of ischemic cardiomyopathy involves a multitude of pathophysiologic mechanisms that determine the extent and severity of the myocardial injury. A major focus of research in this area is to understand cellular mechanisms involved in myocardial insult and try to harness these mechanisms in an effort to diminish the amount of damaged myocardium. In particular it is of major importance to gain better understanding on the physiology and pathophysiology of the Ca^2+^ channel proteins in the heart participating in the global Ca^2+^ homeostasis, under normal conditions and upon cardiac insult. Currently, the most studied Ca^2+^ channel proteins in the cardiomyocytes include the transmembrane L-type channel (Cav1.2), the sarcoplasmic reticulum (SR) SERCA, the ryanodine 2 receptor (RyR2) and the sodium-calcium exchanger (NCX1). Recent data from our laboratory indicate that the newly characterized transient receptor potential Vanilloid 2, TRPV2, channel may also play a role in the pathophysiology of myocardial insult in the setting of acute MI.

TRP channels are a large super-family of non-selective and non-voltage-gated ion channels that convey signaling information linked to a broad range of sensory inputs. They are composed of seven different subfamilies that are related to several physiological and pathological processes [1], [2]. Even though they are nonselective cation channels, most of them are permeable for Ca^2+^ and are gated by diverse stimuli that include intra and extracellular messengers, changes in temperature, chemical and mechanical (osmotic) stress [3]. They have been studied in neurons and inflammatory cells and function as primary sensing molecules in these cell types. In addition these channels have been also shown to be associated with several diseases including cancer and immune diseases [4], [5].

TRPV1 and TRPV2 are the most well studied of these and are known to be very important in the nociception and temperature sensation [6], [7]. TRPV2 is a weak Ca^2+^-selective cation channel known to be activated by swelling of the cells and heat, in addition to specific agonists. This channel consists of six transmembrane regions and is described to be regulated by Insulin-Like Growth Factors- (IGF) [8]. From a cardiovascular point of view, there is growing evidence for the important role of TRP channels in controlling vascular function including endothelial permeability, responses to oxidative stress, myogenic tone, cellular proliferative activity, cellular migration and thermoregulation [9], [10]. TRPV2 is the highest expressed TRPV channel on cardiomyocytes in the murine heart [11], [12]. In addition, recent studies showed that TRPV2 is expressed in phagocyte populations and that the expression of this channel conveys a pivotal role in macrophage particle binding and phagocytosis [7]. It is thus suggested that TRPV2 may harbor a fundamental role in the innate immunity. These data may imply to the potential involvement of TRPV2 in the stormy inflammatory processes taking place upon cardiac ischemia as well as on its potential involvement in the altered Ca^2+^ homeostasis. Nevertheless, the role of TRP channels directly on cardiac function and myocardial ischemia is still unknown.

## Methods

### Ethics statement

All experimental protocols carried out on the animals in this study were approved by the Tel-Aviv Sourasky Medical Center Institutional Review Board (Helsinki Committee) which constitutes our Institutional Animal Care and Use Committee (IACUC).

### Induction of myocardial infarction

Animals were anesthetized with ketamine and xylazine, orally intubated with 22GIV catheter and artificially ventilated with a respirator (Harvard Apparatus). A small oblique thoracotomy was performed lateral to the left intercostal line in the third costal space to expose the heart. Once the pericardium is opened, the proximal left anterior descending artery (LAD) branch of the left coronary artery was permanently ligated using 6–0 polypropylene sutures through a dissecting microscope [13].

### RNA isolation, amplification, labeling and hybridization to DNA chip arrays

RNA from the entire LV tissue of MI (5 days post LAD ligation) or sham-operated controls was extracted by phenol/chlorophorm (Biological industries) and tested for purity by nanodrop and agarose gel electrophoresis. Four samples derived from each one of the two arms were divided to two pools of two RNA samples so that the reactions were started with two pools of two RNA samples in each group (4 samples total). RNA samples were amplified using the Ambion wt expression kit (Applied Biosystems, USA) and hybridized to Affymetrix GeneChip Rat Gene 1.0ST arrays, which interrogates 27,342 genes across 722,254 distinct probes, according to instruction manual. Analysis was performed on CEL files using Partek Genomics Suite TM, version 6.5 Copyright 2010 (http://www.partek.com). Data were normalized and summarized with the robust multi-average method [14].

### Real-time PCR analysis

RNA extracts (500 ng) were transcribed using Verso RT-PCR Kits (ABgene, USA). A quantitative PCR was performed with the Sybr Green PCR kit (Invitrogen, Israel). The primers used for real-time analysis are given in Table 1. The relative mRNA expression of the target genes was normalized to the expression of the GAPDH reference gene.

**Table 1:** Primers and siRNAs used in the study.

	Forward primer	Reverse primer
GAPDH	GGATGCAGGGATGTTC	TGCACCACCAACTGCTTA
TRPV2	GCCGATGGTTTGAAGAG	AGTCAGTGCAGCCCATGGA
IGF-1	GGCATTGTGGATGAGTGTTG	GTCTTGGGCATGTCAGTGTG
**siRNA sequences**		
TRPV2 siRNA 1	AGGAGCUGACUGGACUGCUAGAGUA	
TRPV2 siRNA 2	UACUCUAGCAGUCCAGUCAGCUCCU	
TRPV2 siRNA 3	CCUGCUUUAUUAUACACGUGGCUUU	

### Immunohistochemical (IHC) and immunofluorescence (IFC) staining with CD68 and TRPV2

Isolated LV tissues from sham or MI-induced animals (6 animals/arm) were fixed with 4% paraformaldehyde, sliced into transverse sections and paraffinyzed. The blocks were then sectioned at 4 µm slices. For IHC staining, slides were warmed up to 60°C for 1 h, deparaffinyzed in xylene and rehydrated. An endogenous peroxidase block was performed for 10 min in 3% H_2_O_2_/PBS. For the monocyte/macrophages CD68 (ED-1, Serotec) stain, antigen retrieval was performed with trypsin for 10 minutes, followed by one hour incubation with a 1∶100 diluted mouse anti-rat CD68 antibody. For the TRPV2 staining, after sections were deparaffinyzed and rehydrated, a CC1 standard benchmark pretreatment for antigen retrieval was selected (Ventana Medical Systems). Sections were then incubated one hour at room temperature with Anti-VRL-1 (TRPV2, Novex, 1: 250). Detection was performed with Envision+ System-HRP Labelled Polymer Anti-Rabbit (K4003, Dako). Briefly, sections were incubated for 60 min at room temperature with Envision+ System-HRP Labelled Polymer Anti-Rabbit. The antibody binding was visualized with the substrate-chromogen AEC (Invitrogen Corporation). Sections were counterstained with hematoxylin and cover-slipped with an aqueous mounting fluid (Glycergel, Dako). The stained sections were reviewed with a light microscope and analyzed by a pathologist.

For IFC analysis, antigen retrieval was performed in citrate buffer pH-6.0 for 20 minutes, followed by blocking in CAS buffer (Sigma). The slides were then incubated with mouse anti-rat CD68 (Serotech) diluted 1∶100 in antibody diluent (Rhenium) for one hour. Following washes with PBS, the slides were incubated with Alexa Fluor 488 (AF 488) anti-mouse secondary antibody. Then further washes were applied and the slides were incubated rabbit anti-rat TRPV2 1∶100 (Alamone, acc-039), followed by incubation with anti-rabbit Alexa Fluor 594 (AF 594, Jackson). The slides were then washed and mounted with DAPI Fluoromount-G (Southern Biotech) and analyzed by confocal microscope (Leica SP5).

### Western blot analyses

LV sections from post MI or sham-operated control animals were extracted using a commercial lysis buffer (Sigma). Equal protein amounts (80 µg) were loaded on a 4–20% acrylamide gel followed by electric transfer to a nitrocellulose membranes. Following an overnight blocking with 5% low fat milk diluted in TBS-tween, the membranes were incubated with an anti-insulin-like growth factor 1 (IGF-1) (Santa Cruz, sc-9013), anti-TRPV2 (Alamone, acc-039), anti-TRPV2 pre-incubated with a TRPV2 peptide (Alamone), or with an anti- GAPDH antibody used for validating equal loading (Abcam, clone 6C5). The primary antibodies were followed by blotting with HRP-conjugated secondary antibodies. After rapid incubation with an ECL substrate (Biological Industries, Israel), the membranes were exposed to an imaging film.

### Flow cytometry

Cells were isolated from the LV sections of sham, or post-MI animals at three different time points: 3 day, 10 days and 30 days (6 animals/arm) according to the following protocol: the cardiac tissue was mechanically chopped followed by an enzymatic digestion using collagenase type 2 (Worthington Biochemical Corporation) diluted 1∶100 in HBSS buffer for 30 min at 37°C. Fresh medium was then added in order to terminate the collagenase activity followed by filtration of the cells through 70 µm mesh filters in order to obtain single cell suspensions. The LV-derived cells were then centrifuged and the pellets were incubated with anti-TRPV2-FITC (Alamon lab) and anti-CD11 B/C-PE antibodies (eBioscience) or their corresponding isotype controls, for 1 hour at 4°C in the dark and analyzed by flow cytometry (BD Biosciences FACSCanto II).

### Transient transfection of TRPV2 siRNA to NR8383

NR8383 alveolar macrophages (ATCC clone CRL-2192, USA) were plated in 6 well plates (4*10^5^ cells/well) in the presence of F-12K medium (Biological Industries, Israel) supplemented with 15% heat-inactivated fetal bovine serum, 5% pen/Strep and 5% glutamine. A day later, the cells were transiently transfected with 300 nM TRPV2 siRNA mix composed of three TRPV2 siRNAs (Invitrogen; their sequences are given are Table 1) diluted in 1∶1∶1 ratio optimum medium (Biological Industries, Israel) or with 300 nM of scramble siRNA sequence, using the transfection reagent lipofectamine 2000 (Invitrogen) in a total volume of 1 ml. After 4 hours, the wells were supplemented with 1 ml of F-12K medium with 30% FBS. Sixteen hours later the media were replaced with fresh complete F-12K medium.

### Migration assay

H9C2 myoblast cell line from rat myocardium (ATCC clone CRL-1446) was obtained as a generous gift from Gania Kessler-Icekson, Felsenstein Medical Research Center, Petach Tikva, Israel. The cells were plated in two 6 well plates (1* 10^6^ cells/plate) in complete DMEM medium. One day later the medium was replaced with medium containing 0.3% serum only. Six hours later one of the dishes was placed in a hypoxic chamber containing 1% O_2_ for 18 hours. In parallel, NR8383, were transiently transfected with a mixture of three commercial TRPV2 siRNAs (Invitrogen) or with a scramble-sequence siRNA, as described above. The conditioned media (CM) from both H9C2 plates were collected, centrifuged to get rid of cell debris and divided into 24 wells (600 µl/well). A 3 µM insert (Nunc) was placed above. Fifty thousand NR8383 cells (in 100 µl volume) transfected with TRPV2 siRNA or with a scramble-sequence siRNA were seeded in duplicates on the top of the inserts and were allowed to migrate towards the bottom side if the inserts for 3 hours in a hypoxic chamber at 37°C and 5% CO_2_. The inserts were then taken out, the upper sides were scraped with cotton wool and the cells that migrated to the lower side were fixed with cold ethanol for 10 minutes. The cells were then stained with Commassie blue and destained in water. Photos were immediately taken using Olympus DP73 camera and Cellsense standard 1.6 software. The experiment was repeated twice.

### Statistical analysis

Groups were compared using one-way ANOVA. The Tukey post hoc correction was taken to account for multiple testing (IBM SPSS statistics 20 software). Significance was set at *P*<0.05 (*p<0.05; **p<0.01). Results are expressed as means ± SE.

## Results

### mRNA levels of TRPV2 are significantly upregulated in the cardiac tissue within 3–5 days post-acute MI

**Figure 1:**
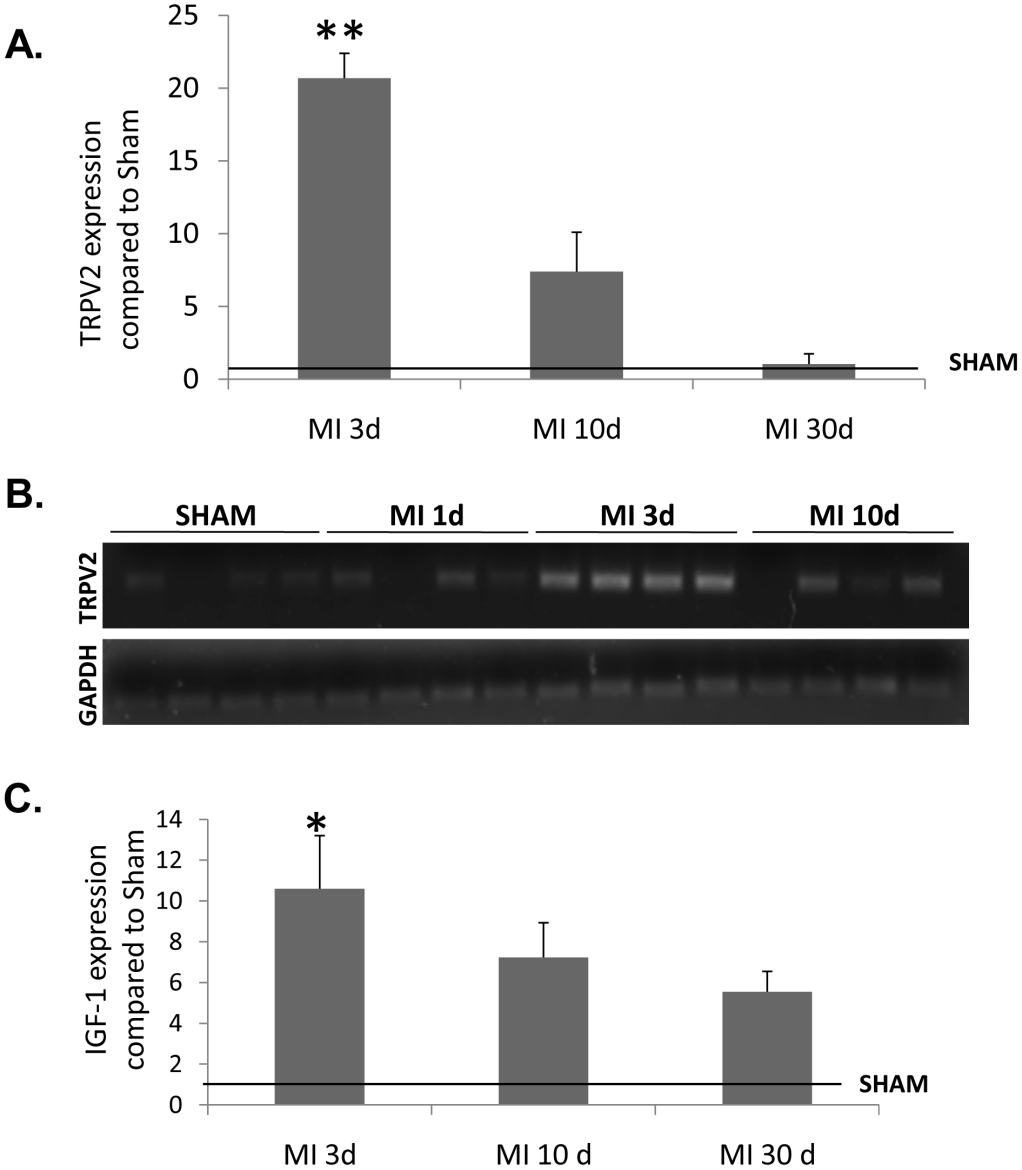
TRPV2 and IGF-1 are overexpressed after acute MI. Real time validation analysis of TRPV2 expression at different time points (3, 10 and 30 days post MI; n = 4/group) is given both quantitatively (A) by calculating the mRNA expression levels using the ΔΔCt method (**p = 0.01) and qualitatively (B) by viewing the PCR products in an agarose gel. (C) Real time analysis for IGF-1 expression in the same mRNA samples used for the real-time analysis of TRPV2 (*p<0.05).

**Figure 2:**
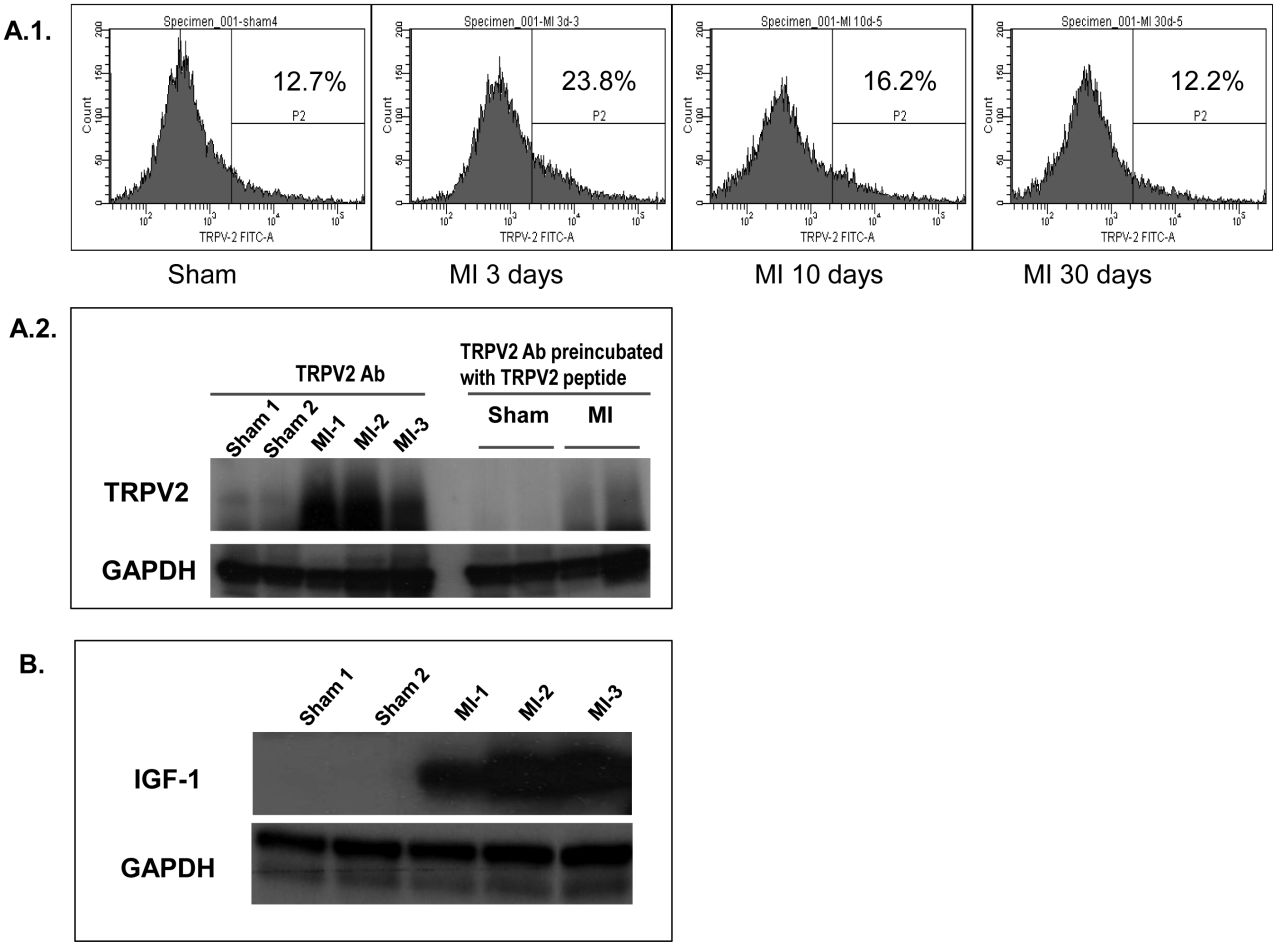
Elevated protein levels of TRPV2 and IGF-1 following acute MI. (A.1) Surface levels of TRPV2 at various time points post MI compared to sham determined by flow cytometry analysis. Cells positive for TRPV2 expression are gated and the percentages are given (A.2). Total TRPV2 protein level three days post MI compared to sham analyzed by Western blot. Left panel of the membrane was blotted with TRPV2 antibody and the right panel was blotted with TRPV2 antibody pre-incubated with a TRPV2 peptide (1∶1 ratio). (B) IGF-1 expression level three days post MI, detected by Western blot analysis.

Using an Affymetrix GeneChip array analysis, we found a clear upregulation of TRPV2 mRNA both in rats 5 days after LAD occlusion (+1.54, p = 0.006, Table 2) and in mice 3 days post procedure (+3.65, p<0.001, Table 3) compared to sham. The expression level of the other members of the TRP gene family including TRPV1, TRPV4, TRPV5 and TRPV6 was not significantly changed relative to the sham samples in both animal models (Tables 2 & 3). A real-time analysis performed on rat RNA samples obtained 3, 10 and 30 days post infarction (4 animals/group), confirmed the marked upregulation of TRPV2 three days post-MI by a fold- change of 20 compared to sham or MI 30 days and 3.5 compared to MI 10 days (p<0.0001, one-way ANOVA). Seven days later (10 days post infarction), TRPV2 expression level was significantly reduced compared to its level on day 3 post infarction (p = 0.0001). Even though at this time point the expression level of the TRPV2 transcripts was still 4- times higher than that of the sham-operated controls, the difference compared to sham was not statistically significant (p = 0.12, one-way ANOVA). Also, no significant differences were observed 30 days post procedure compared to sham (p>0.1; Figure 1A). For qualitative demonstration, PCR products of different cardiac sections derived from sham- operated controls, 1 day, 3 days and 10 days post LAD ligation were loaded onto an agarose gel; a clear upregulation of TRPV2 following an acute MI, especially 3 days post procedure, was evident (Figure 1B). In line with the increased expression of the TRPV2 transcripts, the protein expression on the cell surface of LV-derived cells was significantly increased (p<0.05) as shown in the representative flow cytometry histograms (Figure 2A.1) and Western blot analysis (Figure 2A.2).

**Table 2:** Fold change of several TRP genes (MI versus SHAM, 5 days post procedure) obtained by Affymetrix GeneChip array performed on RNA samples isolated from the LV tissue of rats in an in vivo model for an acute MI.

Gene	Fold-change (MI vs SHAM)	p-value
TRPV1	+1.06	0.17
TRPV2	+1.54	0.006
TRPV4	−1.10	0.11
TRPV5	+1.06	0.20
TRPV6	−1.02	0.70
TRPM1	−1.30	0.60
TRPM2	+1.21	0.20
TRPM7	+1.21	0.30

**Table 3:** Fold change of several TRP genes (MI versus SHAM, 3 days post procedure) obtained by Affymetrix GeneChip array performed on RNA samples isolated from the LV tissue of mice in an in vivo murine model for myocardial infarction.

Gene	Fold-change (MI vs SHAM)	p-value
TRPV1	−1.49	0.0001
TRPV2	+3.65	1*10^−7^
TRPV4	−1.17	0.034
TRPV5	1.00	0.720
TRPV6	−1.10	0.075
TRPM1	−1.13	0.053
TRPM2	+1.30	0.002
TRPM7	+1.29	0.070

A real-time analysis of LV sections of the same experimental animals, revealed that the known regulator of TRPV2, insulin-like growth factor-1 (IGF-1) [15], [16] was upregulated 3-10 days post MI, concomitantly with the overexpression of TRPV2. This upregulation was statistically significant only at the first tested time point, 3 days post infarction (p = 0.007, one-way ANOVA), while the overexpression observed on day 10 or 30 post infarction was not significant (p>0.1; Figure 1C). A Western blot analysis using an anti- IGF-1 antibody confirmed that overexpression of IGF-1 indeed occurs at the protein level three days post MI compared to sham (Figure 2B). The data hint to a signal transduction pathway leading to overexpression of TRPV2 in the peri-infarct macrophages induced by IGF-1.

### TRPV2 is upregulated in macrophages infiltrating to the peri-infarct zone

**Figure 3:**
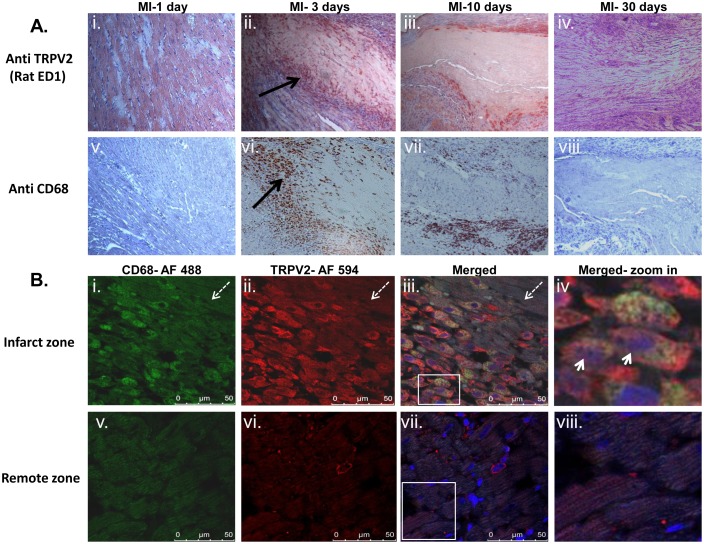
TRPV2 is expressed in the peri-infarct zone of invading macrophages. For IHC analysis (panel A), sections derived from the infarcted zone were stained with either TRPV2 (panels i-iv) or with the monocyte/macrophage marker rat CD68 (panels v-viii). The sections were derived from one of the following time points post LAD occlusion: 1 day (i & v), 3 days (ii & vi), 10 days (iii & vii) and 30 days post procedure (iv & viii). Solid arrows represent positive staining. For IFC analysis (panel B), typical fluorescent images of CD68 AF 488- (green; i & v), TRPV2 AF 594 (red; ii & vi), and merges of the two markers as well as DAPI (iv & viii) are given for LV sections from three days post MI. Panels B i-iv represent the peri-infarct area, while panels v-viii- represent areas remote to the infarct zone, in the same slides. Arrowhead represents several cells stained with both CD68 and TRPV2. The dashed arrows show the infarct zone. The square in panel B iii and vii represent areas that were digitally zoomed in and are given in panels B iv and viii, respectively.

An IHC analysis demonstrated that the TRPV2 staining was exclusively detected in the peri-infarct region 3 days and 10 days post MI induction (Figure 3Aii & iii) but not yet on the first day post infarction (Figure 3Ai) or in LV sections derived from rats that underwent the LAD ligation 30 days earlier (Figure 3A iv) (6 animals/group). A pathologist who was completely blinded to the protocol design suggested that the TRPV2 positive cells are part of the immune system. Since it is widely accepted that 1–7 days post infarction, monocytes invade the infarcted area in significant numbers and maturate into active macrophages, we stained consecutive LV slides with the monocyte/macrophage marker rat CD68 (rat ED-1). Indeed, as expected, almost no macrophages were observed on the first day post LAD ligation or 30 days later (Figure 3A v & viii). A significant accumulation of monocytes/macrophages was observed at the infarcted zone three days post procedure (Figure 3A vi) and to somewhat lower extent seven days later (Figure 3A vii). Part of these CD68-positive macrophages, in particular those ones closest to the infarcted area, also correlated with the positive staining for TRPV2. On the other hand, the LV zones remote from the infarcted area were completely negative for either CD68 or TRPV2 (not shown).

An IFC analysis of slides derived from LV sections from animals three days post MI confirmed that numerous cells in the peri-infarct zone stained positive for CD68 (Figure 3 Bi), TRPV2 (Figure 3 Bii) and that part of the cells dually harbor both CD68 and TRPV2 markers as well as DAPI (Figure 3 Biii and iv). A quantification analysis of the cells which are closest to the infarct zone revealed that 50.2±3.8% of the cells dually stained for CD68 and TRPV2 (not shown). These data point that TRPV2 is highly abundant in macrophages localized in the closest vicinity to the infarct. However, LV areas remote from the infarct zone were negative for both CD68 and TRPV2 (Figure 3B v- viii). All samples stained positive with DAPI, confirming that the infarct area is surrounded by a highly cellular area (Figure 3B iii, iv, vii and viii).

**Figure 4:**
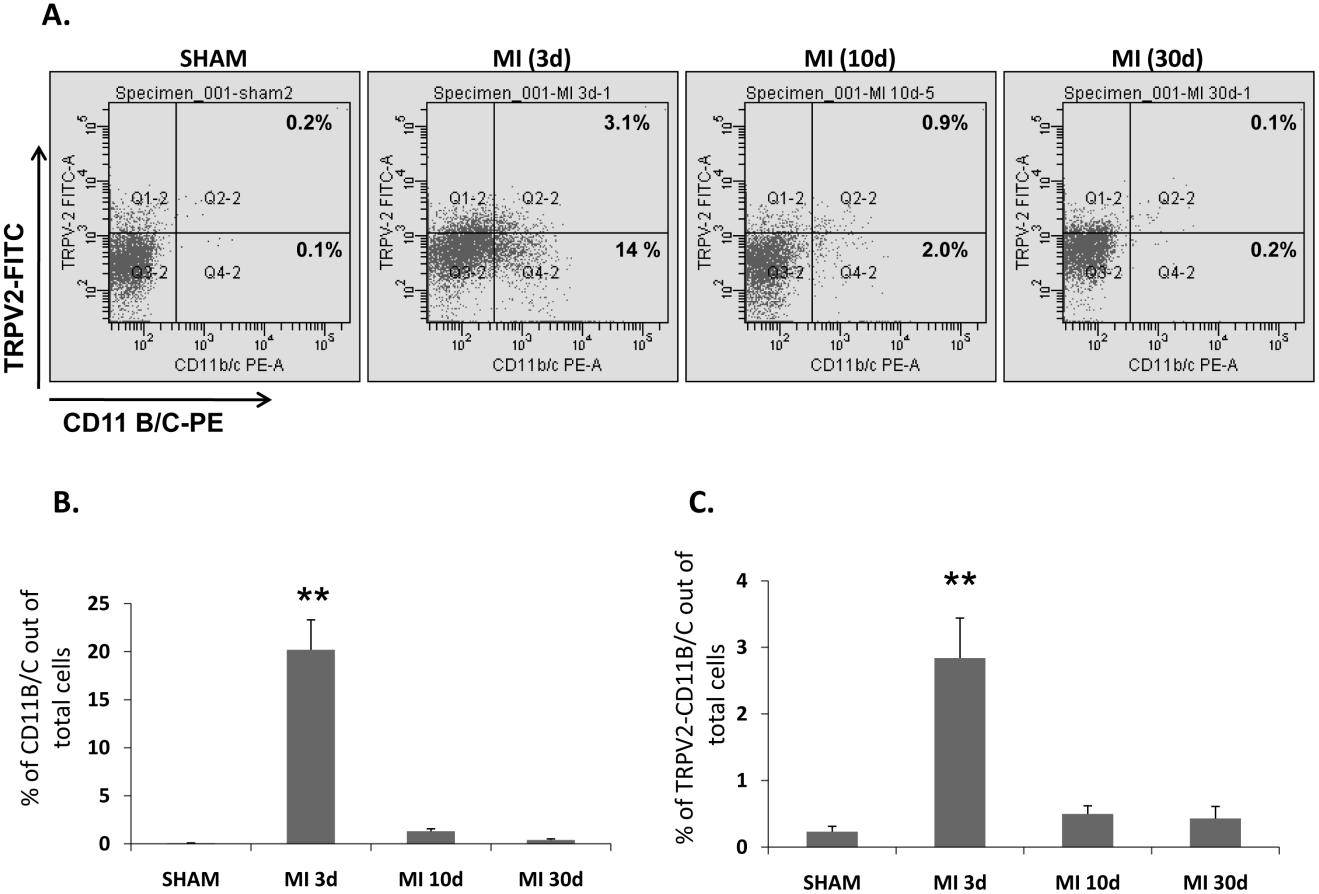
TRPV2 is highly expressed on the surface of peri-infarct macrophages - a flow cytometry analysis. (A) Representative captures of cardiac cells dually expressing CD11B/C and TRPV2 at different time points post MI. Q2-2 upper right quadrates represent the cells which stained positive with both CD11B/C and TRPV2 (TRPV2 expressing monocytes/macrophages). Q4-2 lower right quadrates represent the cells stained positive with CD11B/C only (monocytes/macrophages negative for TRPV2 expression), Q1-2 (upper left quadrates: cells positive for TRPV2 only; Q3-2: cells negative for both CD11B/C and TRPV2. (B) Bar graph showing the percentage of CD11B/C cells out of the total cardiac cells; (C) bar graph demonstrating the percentage of the CD11B/C –TRPV2 dual expressors out of the total cells (6 animals per each time point, **p<0.01).

In order to confirm that TRPV2- elevation upon acute MI is mainly attributed to infiltrating monocytes/macrophages, we employed a flow cytometry analysis on LV-derived cells from experimental animals in which an acute MI was induced for 3 days, 10 days or 30 days (6 animals/group). In line with the IHC and IFC data, the flow cytometry analysis of the LV-derived cells co-stained with the monocyte/dendrytic/granulocyte marker CD11B/C and TRPV2 revealed that three days post MI 20.2±2.4% of the total cardiac cells are infiltrating CD11B/C-positive cells (Figure 4A & B), out of which 15±0.2% express TRPV2 (2.8% of the total cells, Figure 4C). Seven days later, i.e. 10 days post LAD ligation, the percentage of CD11B/C cells went down dramatically to 1.4±0.4%, out of which 36±2.7% (0.6% of the total cells) also expressed TRPV2. As expected, only few monocytes/macrophages were detected in the sham-operated controls (0.3% of the total cells) or in the animals which underwent an acute MI induction 30 days earlier (0.83% of the total cells). Interestingly, it appears that some of these resident CD11B-positive cells harbor TRPV2 expression. Representative figures of the co-staining with CD11B/C and TRPV2 at the various time points is given in Figure 4A. A bar graph of the percentage of the monocytes/macrophages out of the entire LV-derived cells is shown in Figure 4B and the percentage of the CD11B/C-TRPV2 dual-expressor subpopulation out of the total cells is given in Figure 4C.

**Figure 5:**
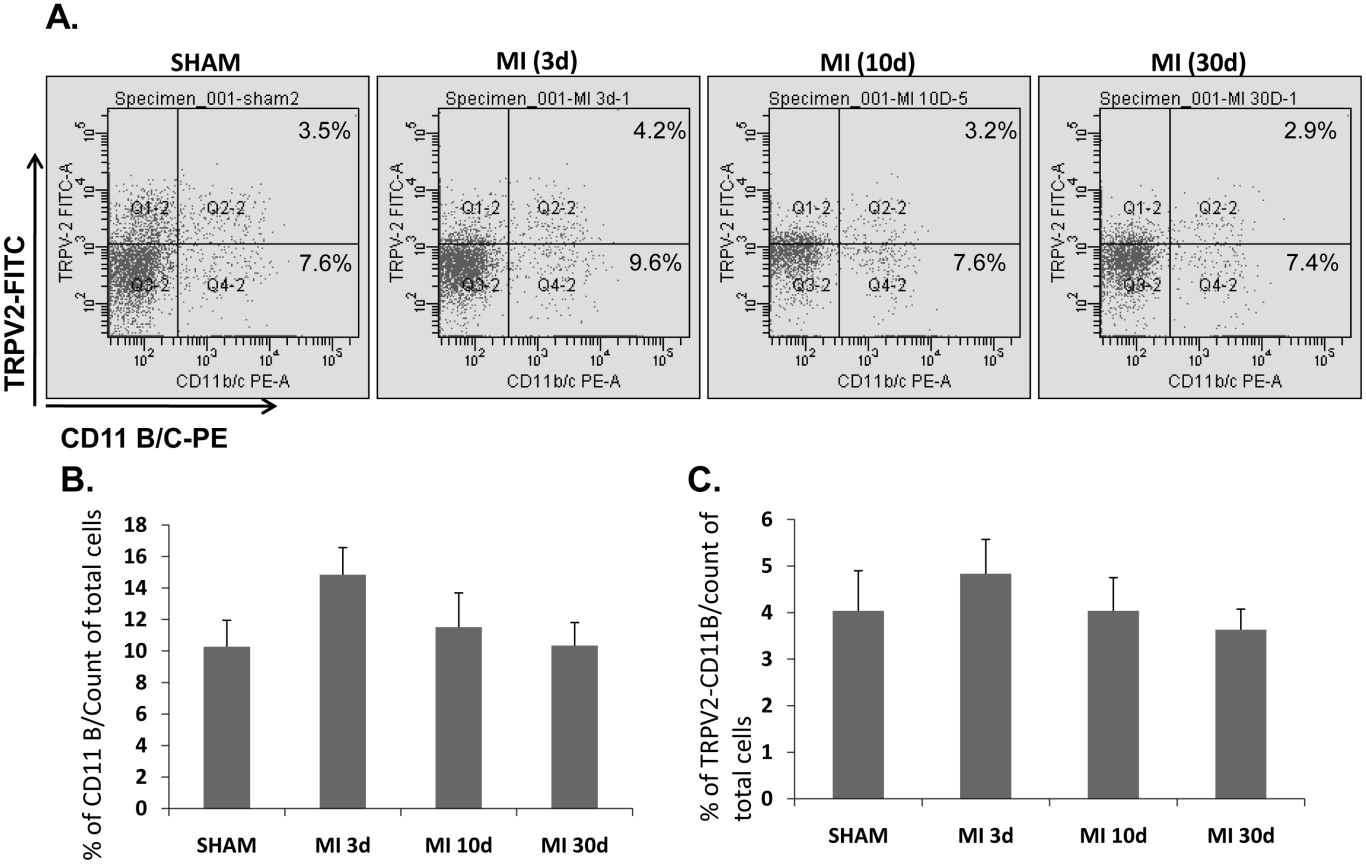
The circulating levels of TRPV2 expressing CD11B/C cells are constant in MI-induced versus sham-operated animals. A flow cytometry analysis showing the percentage of the CD11B/C-expressing cells out of the total circulating cells: (A) Representative capture of cardiac cells dually expressing CD11B/C and TRPV2 at different time points post MI. The four quadrates are designated as described in Figure 3. (B) Bar graph showing the percentage of CD11B/C cells out of the total cardiac cells; (C) bar graph demonstrating the percentage of the CD11B/C –TRPV2 dual expressors out of the total cells. No significant difference was observed between the groups (p>0.5).

Circulating cells isolated from blood withdrawn from the same experimental animals were also double stained with CD11 B/C and TRPV2 and analyzed by flow cytometry. Representative figures of the co-staining of circulating cells of the same experimental animals whose cardiac staining was depicted in Figure 4A, is given in Figure 5A. A bar graph of the percentage of the monocytes/macrophages out of the entire LV-derived cells is shown in Figure 5B and the percentage of the CD11B/C-TRPV2 dual-expressor subpopulation out of the total cells is given in Figure 5C. Interestingly, even though a slight insignificant increase in CD11B/C cells was documented three days post MI compared to the sham animals (Figure 5A & B) as previously suggested [17], overall the percentage of the CD11B/C-TRPV2 double-expressors did not change in the post MI animals compared to sham in all 3 time points (3, 10 or 30 days post MI, p>0.05; Figure 5C). These findings suggest that the upregulation of TRPV2 expression occurs only when the monocytes mature into active macrophages in the ischemic cardiac tissue.

### TRPV2 may be involved in the migration capacity of macrophages towards hypoxic cardiomyocytes

**Figure 6:**
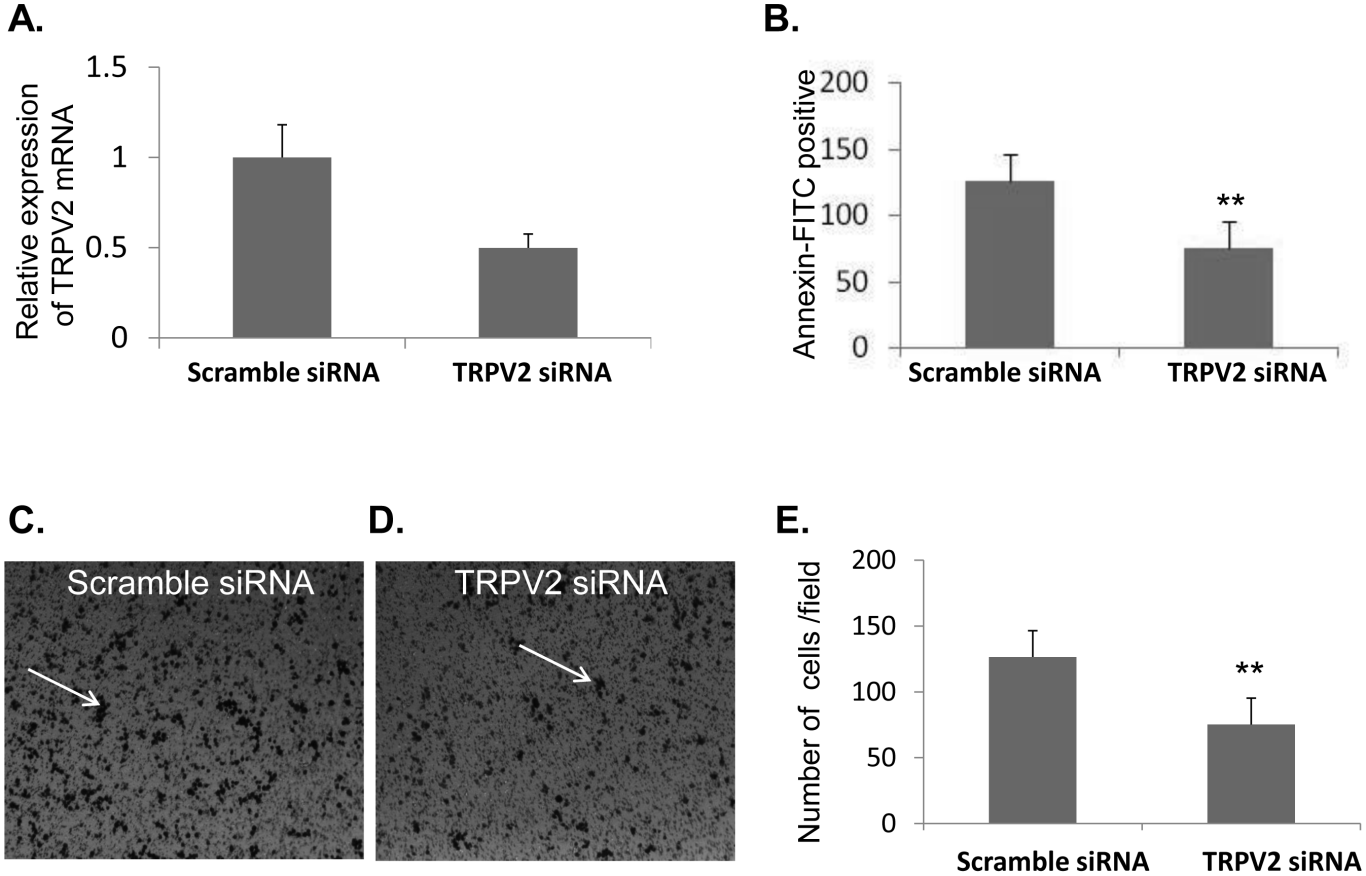
Migration of NR8383 macrophages towards CM of H9C2 cells is TRPV2-dependent under hypoxic conditions. (A) Real-time analysis of TRPV2 expression following transfection with TRPV2 siRNA compared to scramble-control, when normalized to GAPDH. (B) Flow cytometry analysis showing the percentage of early apoptosis (Annexin-FITC positive & PI negative) in H9C2 pre-exposed to hypoxia (**p<0.01). (C & D) Representative captures of NR8383 cells that migrated to the bottom side of the wells, containing hypoxic CM of H9C2, in a transwell migration assay. Migration is shown for NR8383 cells transfected with a scramble siRNA (C) or with a TRPV2 siRNA mix (D). (E) Quantification of the number of migrating NR8383 cells. The experiment was performed in triplicates (3 wells per scramble or TRPV2 siRNA treatment) and 4 different fields in each well were counted (*p = 0.02). Arrows represent migrating cells.

In order to assess whether TRPV2 may affect the migration capacity of macrophages towards ischemic cardiomyocyes, we applied a transewell migration assay in which NR8383 macrophages were allowed to migrate towards conditioned media of hypoxic H9C2 cells for 4 hours. First, we verified a 50% downregulation in TRPV2 expression in the NR8383 cells following transient transfection with the TRPV2 siRNA (Figure 6A). In addition we observed that the 18 hour exposure of H9C2 cells to 1% O_2_ resulted in a significant increase in the number of apoptotic cells compared to the control cell culture (18.2±1.3% versus 30.6±1.3% early apoptosis in hypoxic versus normoxic H9C2, Figure 6B), implying that H9C2 cells are sensitive to hypoxic conditions. The results of the migration assay demonstrated that transfection with TRPV2 siRNA leads to a significant reduction in the number of the migrating macrophage cells towards CM of hypoxic myoblasts compared to macrophages transfected with mock siRNA. Representative captures of NR8383 that migrated to the bottom side of the inserts following transfection with scramble siRNA or TRPV2 siRNA are given in Figure 6C and 6D, respectively. A bar graph of the average number of migrating cells counted in 4 fields in two duplicate wells per treatment in two independent experiments (4 wells total) is shown in Figure 6E. The data suggest that TRPV2 may be involved in mediating the migration and perhaps the subsequent phagocytosis of injured cardiomyocytes by macrophages accumulating around the infarct area.

## Discussion

The early stage of myocardial infarction involves massive infiltration of inflammatory cells mainly into the peri-infarct region [18]. Macrophages play a role in the removal of the necrotic tissue and in the initiation of a healing process. In the present study we show that the cation channel TRPV2 is significantly overexpressed in part of the peri-infarct macrophages. This elevation is exclusive to TRPV2 since it was not observed in any other member of the TRP gene superfamily. Interestingly, no modification in TRPV2 expression was observed in circulating monocytes, implying that the increased transcription of the TRPV2 gene occurs locally in the ischemic zone, concomitantly with monocyte maturation to active macrophages around the infarction zone. TRPV2, a divalent cation channel, is highly expressed in immune related organs including the spleens; however TRPV2 transcripts have also been detected in many resident macrophage populations such as liver Kupffer cells, skin epidermal Langerhans cells and lung alveolar macrophages. TRPV2 expression has also been identified in mast cell populations and in lymphocytes [19]. In this study we have shown that the cardiac resident macrophages also express TRPV2.

Recent literature indicates that TRPV2 may play a crucial role in innate immunity processes [8], [15], [20]. The reported data suggested that under unstimulated conditions, TRPV2 is localized abundantly in the intracellular compartments and that upon ligand stimulation, TRPV2 translocates to the plasma membrane by a phosphatidylinositol (PI) 3 kinase-dependent mechanism. They also suggested that the TRPV2 translocation to the plasma membrane may regulate the cytoskeletal organization necessary for phagocytic receptor clustering, known as the macrophage podosome. The phagosome eventually enhances the migration capacity of the macrophages by regulating the increase in Ca^2+^ entry. Later on, using TRPV2 deficient mice, Caterina and his colleagues have shown that phagocytic processes are impaired in macrophages lacking this cation channel [7]. We thus assume that the significant elevation in TRPV2 expression in cardiac macrophages may harbor a significant role in post MI innate immunity leading to wound healing and scar formation.

Myocardial infarction causes sterile inflammation, which is characterized by recruitment and activation of innate and adaptive immune system cells, as a prerequisite for healing and scar formation. It is currently established that monocytes infiltrate the infarcted myocardium in increasing numbers over the first week post MI [21]. After recruitment in the infarcted territory, monocytes differentiate into macrophages. While macrophages are functionally heterogeneous, it is established that the first responders dominating up to 5 days post infarction, usually referred to as M1- macrophages, exhibit high expression levels of proinflammatory mediators and may play a role in early phagocytosis of the damaged cardiomyocytes [22].

It has been demonstrated that IGF-1 may act as a potential ligand for TRPV2 in vivo. Recent data show that when cells are stimulated with IGF-1, a relatively large amount of TRPV2 is recruited from the intracellular pool and translocates to the plasma membrane. As a result, the expression of TRPV2 in the plasma membrane increases considerably and calcium entry is augmented. When the action of IGF-1 is removed, the supply of TRPV2 from the intracellular pool is terminated and the TRPV2 expressed on the plasma membrane undergoes endocytosis and returns to the endoplasmic reticulum. Consequently, the amount of TRPV2 expressed in the plasma membrane is reduced gradually. In line with our data presented herein, it has been established that shortly after LAD ligation the expression levels of IGF-1 in the cardiomyocytes is elevated [23], [24]. Moreover, it has been demonstrated that the cardiac Ca^2+^ channel activity is augmented by exposure to IGF-1 and that shortly after LAD ligation, the expression levels of IGF-1and IGF-1 receptor are elevated and thus activate the IGF-1/IGF-R autocrine signaling pathway [23]. Interestingly, it has been reported that while shortly after an acute MI IGF-1 peptide is mainly localized at the cardiomyocytes in surviving tissue, later on IGF-1 peptide becomes detectable in macrophages infiltrating the repair zone [25]. We therefore hypothesize that, in addition to its autocrine activity, IGF-1 secreted from the injured cardiomyocytes, may harbor paracrine activity that induces intracellular trafficking of TRPV2 to the plasma membrane in the adjacent macrophages. We assume that IGF-1 binding to TRPV2 molecules on the peri-infarct macrophages, may initialize a signaling cascade that activates PI-3 kinase activity, which is in turn responsible for the translocation of TRPV2 to the plasma membrane, as previously suggested [15]. This paracrine activity probably affects best the macrophages which are closest to the infarct zone. This hypothesis may account for the data observed in the immnohistochemical analysis showing that three days post myocardial infarction, TRPV2 is expressed mainly in those macrophages which are closest to the injured cardiomyocytes in the peri-infarct zone.

**Figure 7:**
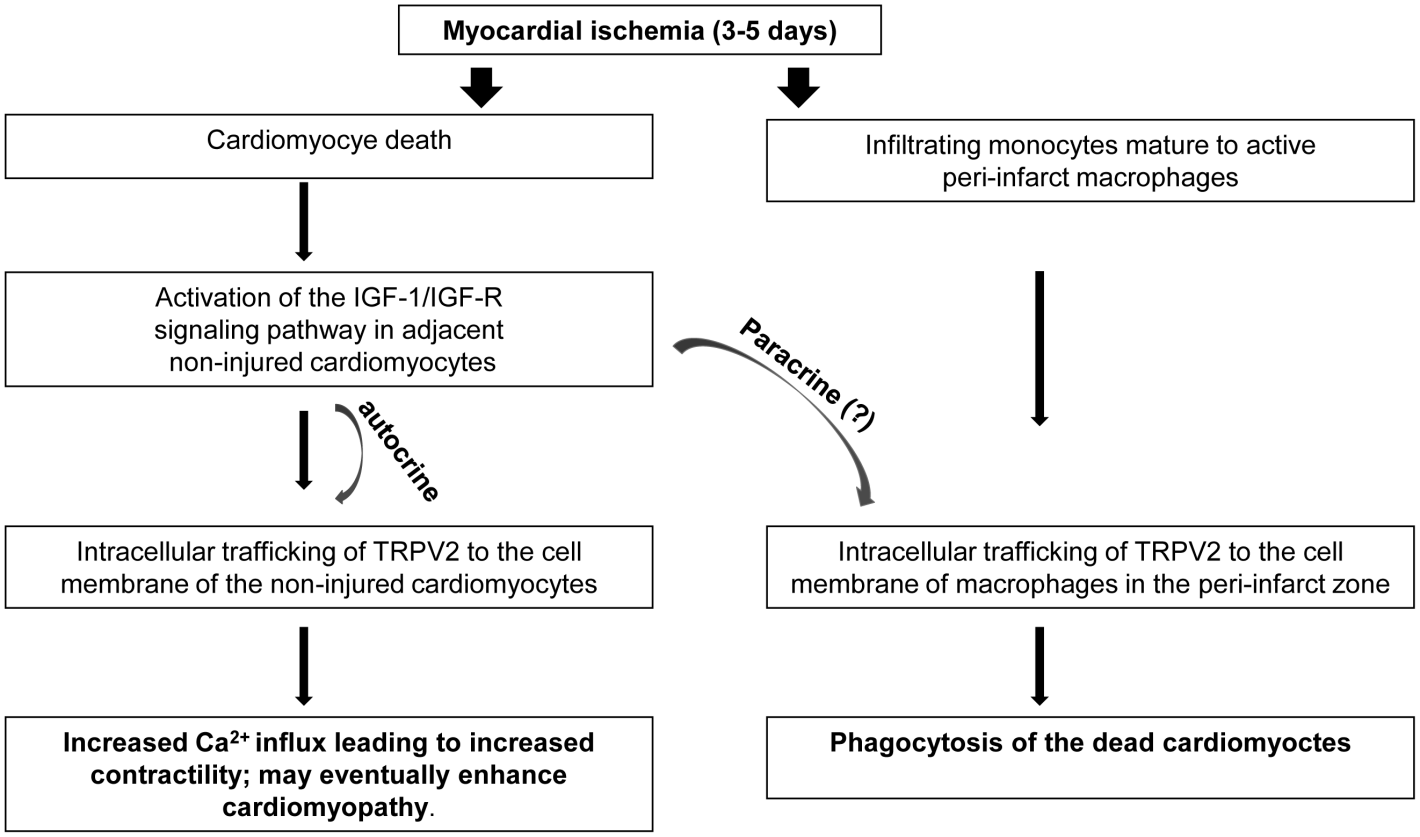
Proposed scheme for the potential IGF-1/TRPV2 crosstalk. Shortly after myocardial infarction IGF-1 leads to TRPV2 trafficking to the cell membrane of cardiomyocytes through the autocrine signaling pathway as previously described [23]. Paracrine effects of IGF-1 on peri-infarct macrophages may also take place as it has been reported that IGF-1 peptide becomes detectable several days post MI in macrophages infiltrating the repair zone [25]. IGF-1 may thus enhance TRPV2 trafficking to the cell membrane of adjacent macrophages via paracrine effects.

In addition, the data presented suggest that the migration capacity of the activated peri-infarct macrophages towards the injured cardiomyocytes is probably, at least in part, TRPV2 dependent, since it was significantly inhibited when TRPV2 expression was downregulated by siRNA. A proposed scheme for the mode of TRPV2 involvement in the phagocytosis of the injured cardiomyocytes shortly after acute MI, is given in Figure 7.

Study limitations: in this study we have focused on the potential involvement of TRPV2 in the immune reactions occurring following an acute MI. Nevertheless, published evidence suggests that dilated cardiomyopathy, a severe disorder defined by ventricular dilation and contractile dysfunction, is associated with an accumulation of TRPV2 in cardiomyocytes in various heart failure animal models and in patients [26]. On the other hand, recent data suggest that TRPV2 elimination from murine hearts results in severe cardiac dysfunction accompanied by disorganization of the intercalated discs that support mechanical coupling with neighboring cardiomyocytes [27]. Therefore, the potential roles of the cardiomyocyte-TRPV2 in cardiomyocyte loss should also be studied thoroughly at various time points post infarction. In addition, in the current study we have utilized a model for permanent LAD occlusion. It may be interesting to examine the TRPV2 expression levels in infiltrating macrophages in an ischemia/reperfusion model which may mimic better the clinical presentation of most patients who undergo cardiac catheterization procedure shortly after the experiencing the symptoms of acute MI. Furthermore, the exact roles of TRPV2 in the post MI healing processes in general and in post MI innate immunity in particular including the possible effects of IGF-1 on TRPV2 expression on macrophages, are still unclear. To this end, we intend to perform an additional set of in vivo experiments in which we will analyze cardiac function and pathology after MI induction in whole- body TRPV-2 knockout mice [7] with or without adoptive transfer of TRPV2 expressing macrophages. Moreover, further in vivo studies involving overexpression/depletion of IGF-1, may provide an insight to the potential IGF-1- TRPV2 paracrine crosstalk.

### Conclusions

The data presented herein demonstrate that TRPV2 expressing macrophages may play a significant role in the inflammatory processes that occur after permanent LAD occlusion at the local environment of the infarcted LV. The TRPV2 gene overexpression and trafficking of the mature channel protein to the macrophage cell membrane may enhance the phagocytic activity of the peri-infarct macrohages, as suggested by the in vitro migration assay. Taken together, the data obtained may provide a better insight into the function of TRPV2 expressed on the cell surface of the post-MI infiltrating macrophages. The data may, thus, lead to the identification of a novel therapeutic target for patients experiencing an acute MI.
